# Hans-Dieter Klimm, Frank Peters-Klimm: Allgemeinmedizin: Der Mentor für die Facharztprüfung und für die allgemeinmedizinische ambulante Versorgung

**DOI:** 10.3205/zma001705

**Published:** 2024-11-15

**Authors:** Lisa Schumacher, Marco Roos

**Affiliations:** 1 Universität Augsburg, Lehrstuhl für Allgemeinmedizin, Augsburg, Germany

## Bibliographical details

Hans-Dieter Klimm, Frank Peters-Klimm

**Allgemeinmedizin: Der Mentor für die Facharztprüfung und für die allgemeinmedizinische ambulante Versorgung**

Publisher: Georg Thieme Verlag, Stuttgart

Year of publication: 2023, 853 pages, price: € 129.99

ISBN: 978-3-13-243593-3

## Review

### Summary

The seventh and revised edition of “Allgemeinmedizin: Der Mentor für die Facharztprüfung und für die allgemeinmedizinische ambulante Versorgung” by editors Hans-Dieter Klimm and Frank Peters-Klimm was published by Georg Thieme Verlag in 2023. The book is divided into five parts and is logically structured. First, the authors provide information on the structure of specialty training in general practice and conclude their explanations with chapters for daily routine. With over 800 pages, the book is comprehensive and covers all relevant areas of general practice. It takes postgraduate trainees by the hand and accompanies them through to the final examination, but also serves as a reference work for general practitioners.

### Versatility of general practice 

The book “Allgemeinmedizin” has been accompanying postgraduate trainees and general practitioners since 1996. Since then, innovations and changes, not only in the field of medicine but also in relation to legal circumstances, have repeatedly found their way into the current editions, and so all the contents of the seventh edition have also been revised and updated. The addition of chapters on video consultations, digitalization and additional clinical pictures reflect relevant developments in health services. The team of editors and authors is made up of general practitioners and colleagues working at universities and thus offers a wide range of views on the subject.

In the first part, general practice is defined by the German College for General Practice and Family Medicine (DEGAM) and the Federal Doctors Chamber and its areas of work are presented. In addition, the structure of specialty training up to the final examination is outlined and the working principles of general practice are discussed. Part two is dedicated to prevention. Primary prevention includes vaccination recommendations and information on travel medicine as well as health education and health advice. In secondary prevention, attention is paid to preventive care for children with a tabular presentation of preventive check-ups for children and adolescents. For adults, general health check-ups and early cancer detection are presented. In the “reason for encounter – from symptom to diagnosis” section of the third part, readers learn about the most common reasons for consultation in adults and children and the most common combinations of illnesses, divided into the subsections pain, complaints and leading symptoms. Each possible reason for consultation is subdivided into definition and epidemiology, diagnostic considerations and further diagnostic testing. An overview of therapeutic methods in practice can be found in chapter four. This includes not only drug therapy, psychotherapy, nutritional therapy and exercise therapy, but also complementary medicine. Furthermore, the authors address the role of general practitioners in special therapeutic procedures, such as pressure ulcer treatment or tumor aftercare. In the fifth and final part, the most important and most common clinical pictures are presented in alphabetical order from A for abortion to Z for cytomegaly. Each clinical picture is first defined, followed by information on epidemiology, etiology, leading symptoms, diagnostics and differential diagnostics. Treatment options are outlined, in some cases with dosage recommendations. It should be noted that COVID-19 diseases are not mentioned in this chapter or the entire book due to the current rapidly changing state of knowledge.

The book is clearly structured. The chapters and sub-chapters are marked in the margins of the pages for quick reference. Cross-references to other chapters within the book allow the information to be linked. Beginning with the general introduction to specialty training and the workflow in general practice through, the book follows a logical structure in which it becomes increasingly specific to everyday practice and thus to dealing with patients and their concerns. “Allgemeinmedizin” is largely based on texts that are easy to understand and are limited to core information. Key points are often used to provide a quick overview of the topic in question. In addition, tables and illustrations present more complex contexts clearly. Colored boxes, strong eyecatcher, are used to draw attention to dangerous processes that can be avoided or to point out special features under “note”. As the structure of the chapters always follows the same pattern, the structured approach to finding a diagnosis and the appropriate treatment is reinforced. However, the reference to clinical guidelines and further literature offers the opportunity to delve deeper into the relevant topic. The fact that the book can be unlocked on the eRef online platform using a personal access code means that it can be read on smartphones or PCs, making it easy to access on the move. Increasingly, providers of practice management systems are also offering a direct link between eRef and their services, which makes it easier to integrate specialist literature into everyday working life. 

In conclusion, “Allgemeinmedizin: Der Mentor für die Facharztprüfung und für die allgemeinmedizinische ambulante Versorgung“ is a successful work that manages the balancing act between a workbook for general practice and a medical reference work for general practitioners at various stages of their careers, bundling information from different areas and presenting it in a structured way. The focus on the essentials makes it a good reference book and revision guide. If you have little prior knowledge, we recommend reading the book together with other sources, as basic knowledge is assumed, especially in the field of pathophysiology. At a price of over 120 euros, the book is not inexpensive, but the scope certainly justifies the price.

## Author’s ORCID

Marco Roos: [0000-0003-1596-5908]

## Competing interests

The authors declare that they have no competing interests. 

